# Conformational changes associated with the binding of zinc acetate at the putative active site of *Xc*TcmJ, a cupin from *Xanthomonas campestris* pv. *campestris*

**DOI:** 10.1107/S1744309109021988

**Published:** 2009-10-27

**Authors:** Herbert L. Axelrod, Piotr Kozbial, Daniel McMullan, S. Sri Krishna, Mitchell D. Miller, Polat Abdubek, Claire Acosta, Tamara Astakhova, Dennis Carlton, Jonathan Caruthers, Hsiu-Ju Chiu, Thomas Clayton, Marc C. Deller, Lian Duan, Ylva Elias, Julie Feuerhelm, Slawomir K. Grzechnik, Joanna C. Grant, Gye Won Han, Lukasz Jaroszewski, Kevin K. Jin, Heath E. Klock, Mark W. Knuth, Abhinav Kumar, David Marciano, Andrew T. Morse, Kevin D. Murphy, Edward Nigoghossian, Linda Okach, Silvya Oommachen, Jessica Paulsen, Ron Reyes, Christopher L. Rife, Henry J. Tien, Christina V. Trout, Henry van den Bedem, Dana Weekes, Aprilfawn White, Qingping Xu, Chloe Zubieta, Keith O. Hodgson, John Wooley, Marc-André Elsliger, Ashley M. Deacon, Adam Godzik, Scott A. Lesley, Ian A. Wilson

**Affiliations:** a Joint Center for Structural Genomics, http://www.jcsg.org, USA; b Stanford Synchrotron Radiation Lightsource, SLAC National Accelerator Laboratory, Menlo Park, CA, USA; c Program on Bioinformatics and Systems Biology, Burnham Institute for Medical Research, La Jolla, CA, USA; d Protein Sciences Department, Genomics Institute of the Novartis Research Foundation, San Diego, CA, USA; e Center for Research in Biological Systems, University of California, San Diego, La Jolla, CA, USA; f Department of Molecular Biology, The Scripps Research Institute, La Jolla, CA, USA; g Photon Science, SLAC National Accelerator Laboratory, Menlo Park, CA, USA

**Keywords:** zinc-binding sites, conformational changes, metalloproteins, ligand binding, structural genomics

## Abstract

In the plant pathogen *Xanthomonas campestris* pv. *campestris*, the product of the *tcmJ* gene, *Xc*TcmJ, encodes a protein belonging to the RmlC family of cupins. *Xc*TcmJ was crystallized in a monoclinic space group (*C*2) in the presence of zinc acetate and the structure was determined to 1.6 Å resolution. Previously, the apo structure has been reported in the absence of any bound metal ion [Chin *et al.* (2006 ▶), *Proteins*, **65**, 1046–1050]. The most significant difference between the apo structure and the structure of *Xc*TcmJ described here is a reorganization of the binding site for zinc acetate, which was most likely acquired from the crystallization solution. This site is located in the conserved metal ion-binding domain at the putative active site of *Xc*TcmJ. In addition, an acetate was also bound within coordination distance of the zinc. In order to accommodate this binding, rearrangement of a conserved histidine ligand is required as well as several nearby residues within and around the putative active site. These observations indicate that binding of zinc serves a functional role in this cupin protein.

## Abstract 2

The crystal structure of an RmlC-type cupin with zinc acetate bound at the putative active site reveals significant differences from a previous structure without any bound ligand. The functional implications of the ligand-induced conformational changes are discussed.

## 1. Introduction

**Figure 1:**
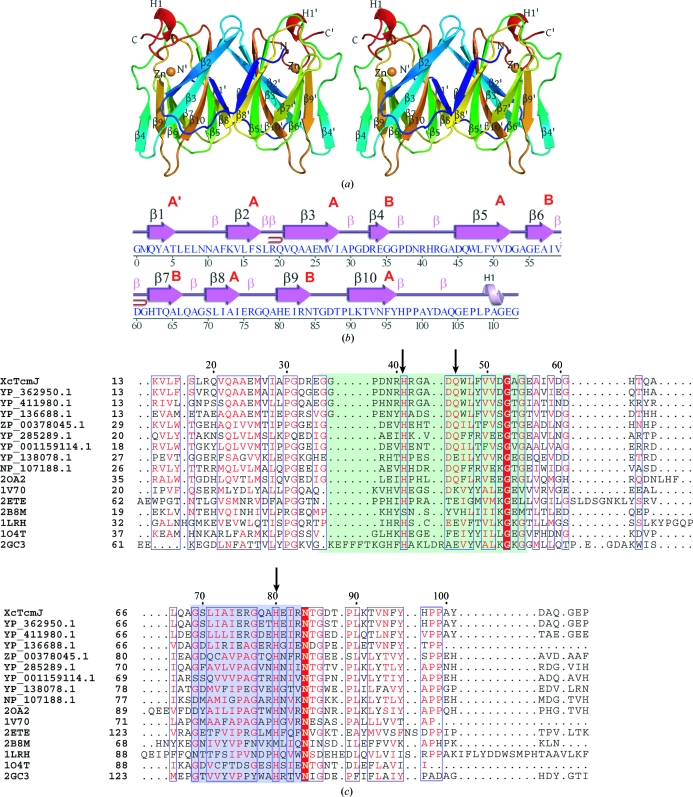
Crystal structure of *Xc*TcmJ from *X. campestris* pv. *campestris*. (*a*) Stereo ribbon diagram of the *Xc*TcmJ dimer color coded from the N-terminus (blue) to the C-terminus (red). Helices H1 and H1′ and β-strands (β1–β10, β1′–β10′) are indicated. A Zn atom in the vicinity of the putative active site of each monomer is shown as a sphere (orange). (*b*) Diagram showing the secondary-structure elements of *Xc*TcmJ superimposed on its primary sequence. The 3_10_-helices, β-strands and β-­turns are indicated. The β-sheets are indicated in red letters. The β-hairpins are depicted as red loops. (*c*) Multiple sequence alignments for *Xc*TcmJ and related proteins. The first four protein sequences belong to *Xc*TcmJ (GenBank NP_636471) and its putative orthologs (GenBank YP_362950, YP_411980 and YP_136688). Sequences 5–16 belong to proteins that are similar but have a different pattern of conserved residues (GenBank or PDB codes ZP_00378045, YP_285289, YP_001159114, YP_138078, NP_107188, 2oa2, 1v70, 1o4t, 1lrh and 2gc3). The positions of the three residues (His41, Gln46 and His80) within coordination distance of the Zn atom in the putative active site in the *Xc*TcmJ structure are labeled with arrows. The two conserved cupin sequence motifs (Dunwell *et al.*, 2004 ▶) are shaded in green and blue.

The *tcmJ* gene (Shen & Hutchinson, 1993 ▶) of *Xanthomonas cam­pestris* pv. *campestris* strain ATCC 33913 encodes a protein from the cupin superfamily, *Xc*TcmJ, with a molecular weight of 12.2 kDa (residues 1–113) and a calculated isoelectric point of 4.9. *Xc*TcmJ was selected for structure determination in order to extend the structural coverage of proteins of unknown function from pathogenic bacteria, in this case *X. campestris* pv. *campestris* (Leyns *et al.*, 1984 ▶). This Gram-negative bacterium causes black rot, which is one of the major worldwide diseases of cruciferous crops (Crossman & Dow, 2004 ▶; Williams, 1980 ▶). In *X. campestris* pv. *campestris*, a variety of poly­ketide metabolites are synthesized which are precursors of antibiotics and antitumor therapeutics (Donadio *et al.*, 2007 ▶; Tudor-Nelson *et al.*, 2003 ▶). *Xc*TcmJ is among several gene products from *X. campestris* that have been annotated as being involved in tetracenomycin polyketide biosynthesis (Overbeek *et al.*, 2005 ▶). A sequence-database search found four proteins that are orthologous to *Xc*TcmJ (Fig. 1 ▶ *c*), as well as over 1000 more distantly related proteins from several different protein families.

*Xc*TcmJ belongs to the cupin superfamily, the members of which have a conserved β-barrel fold and have been classified into at least 18 functional classes involved in a range of biochemical processes, including plant growth and development (Dunwell *et al.*, 2001 ▶, 2004 ▶). In enzymatic cupins, the nature of the bound metal cofactor is believed to influence the type of reaction catalyzed. Although some characteristics of the metal ion-binding motif are conserved across all members of the cupin superfamily, several crystal structures of enzymatic cupins have been reported without any bound metal cofactor.

Comparison of apo and metal-bound cupin structures should, therefore, enhance our understanding of how metal-ion binding influences functional specificity in this superfamily of proteins. Since no metal ion was found in the previously solved crystal structure of an identical protein from a different strain (XC5357 from *X. campestris* pv. *campestris* strain 17; PDB code 2gu9; Chin *et al.*, 2006 ▶), it was speculated that XC5357 may not require a metal ion for activity. Here, we report the crystal structure of *Xc*TcmJ from *X. campestris* pv. *campestris* with zinc acetate bound at the putative active site at 1.6 Å resolution which offers additional insights into the role of metal ions in the structure and function of cupins. The structure was determined using the semi-automated high-throughput pipeline of the Joint Center for Structural Genomics (JCSG; Lesley *et al.*, 2002 ▶) as part of the National Institute of General Medical Sciences Protein Structure Initiative (PSI; http://www.nigms.nih.gov/Initiatives/PSI/).

## 2. Materials and methods

### 2.1. Protein production and crystallization

The gene encoding *Xc*TcmJ (GenBank NP_636471.1; gi:21230554) was amplified by polymerase chain reaction (PCR) from genomic DNA using *PfuTurbo* DNA polymerase (Stratagene) and primers corresponding to the predicted 5′ (aacctgtacttccagggcATGCAGTA­CGCAACGTTGGA) and 3′ (gagttaattaagtcgcgttaGCCTTCGCCT­GCCGGCAA) ends. The PCR product was cloned into plasmid pSpeedET, which encodes an expression and purification tag followed by a tobacco etch virus (TEV) protease cleavage site (MGSDKIHHHHHHENLYFQ/G) at the amino-terminus of the full-length protein. The cloning junctions were confirmed by DNA sequencing. Protein expression was performed in selenomethionine-containing medium with suppression of normal methionine synthesis using *Escherichia coli* strain GeneHogs (Invitrogen). At the end of fermentation, lysozyme was added to the culture to a final concentration of 250 µg ml^−1^ and the cells were harvested. After one freeze–thaw cycle, the cells were sonicated in lysis buffer [50 m*M* HEPES pH 8.0, 50 m*M* NaCl, 10 m*M* imidazole, 1 m*M* tris(2-carboxyethyl)­phosphine hydrochloride (TCEP)]. The lysate was clarified by centrifugation at 32 500*g* for 30 min and loaded onto nickel-chelating resin (GE Healthcare) pre-equilibrated with lysis buffer. The resin was washed with wash buffer [50 m*M* HEPES pH 8.0, 300 m*M* NaCl, 40 m*M* imidazole, 10%(*v*/*v*) glycerol, 1 m*M* TCEP] and the protein was eluted with elution buffer [20 m*M* HEPES pH 8.0, 300 m*M* imidazole, 10%(*v*/*v*) glycerol, 1 m*M* TCEP]. The eluate was buffer-exchanged with HEPES crystallization buffer (20 m*M* HEPES pH 8.0, 200 m*M* NaCl, 40 m*M* imidazole, 1 m*M* TCEP) and treated with 1 mg of TEV protease per 15 mg of eluted protein. The digested protein was passed over nickel-chelating resin (GE Healthcare) pre-equilibrated with HEPES crystallization buffer and the resin was washed with the same buffer. The flowthrough and wash fractions were combined and concentrated to 18 mg ml^−1^ by centrifugal ultrafiltration (Millipore) for crystallization assays. *Xc*TcmJ was crystallized using the nanodroplet vapor-diffusion method (Santarsiero *et al.*, 2002 ▶) with standard JCSG crystallization protocols (Lesley *et al.*, 2002 ▶). Sitting drops composed of 200 nl protein mixed with 200 nl crystallization solution were equilibrated against a 50 µl reservoir at 277 K for 34 d prior to harvesting. Initial screening for diffraction was carried out using the Stanford Automated Mounting system (SAM; http://smb.slac.stanford.edu/facilities/hardware/SAM/UserInfo; Cohen *et al.*, 2002 ▶) at the Stanford Synchrotron Radiation Lightsource (SSRL). The crystallization reagent contained 35%(*v*/*v*) 2-propanol, 0.2 *M* zinc acetate and 0.1 *M* imidazole pH 8.0. No additional cryoprotectant was added to the crystal. Diffraction data from a cube-shaped crystal of approximate dimensions 100 × 100 × 100 µm mounted in a nylon loop were indexed in monoclinic space group *C*2 (Table 1 ▶). The molecular weight and oligomeric state of *Xc*TcmJ were determined using a 1 × 30 cm Superdex 200 column (GE Healthcare) in combination with static light scattering (Wyatt Technology). The mobile phase con­sisted of 20 m*M* Tris pH 8.0, 150 m*M* NaCl and 0.02%(*w*/*v*) sodium azide.

### 2.2. Data collection, structure solution and refinement

Multi-wavelength anomalous diffraction (MAD) data were collected at SSRL on beamline BL11-1 at wavelengths corresponding to the high-energy remote (λ_1_), inflection (λ_2_) and peak (λ_3_) of a selenium MAD experiment. The data sets were collected at 100 K using an ADSC Quantum 315 CCD detector. The MAD data were integrated and scaled using the *XDS* package (Kabsch, 1993 ▶). Data statistics are summarized in Table 1 ▶. The selenium substructure was solved with *SHELXD* (Sheldrick, 2008 ▶) and the MAD phases were refined with *autoSHARP* (Vonrhein *et al.*, 2007 ▶), which resulted in a mean figure of merit of 0.42 with two selenium sites. Automated model building was performed by iterative cycles of *ARP*/*wARP* (Cohen *et al.*, 2004 ▶). Model completion and refinement were performed using *Coot* (Emsley & Cowtan, 2004 ▶) and *REFMAC* (Winn *et al.*, 2003 ▶). Refinement statistics are summarized in Table 1 ▶.

### 2.3. Validation and deposition

The quality of the crystal structure was analyzed using the *JCSG Quality Control* server, which verifies the stereochemical quality of the model using *AutoDepInputTool* (Yang *et al.*, 2004 ▶), *MolProbity* (Lovell *et al.*, 2003 ▶) and *WHATIF* 5.0 (Vriend, 1990 ▶), the agreement between the atomic model and the data using *SFCHECK* 4.0 (Laskowski *et al.*, 2005 ▶) and *RESOLVE* (Terwilliger, 2002 ▶), the protein sequence using *ClustalW* (Chenna *et al.*, 2003 ▶), the atom occupancies using *MOLEMAN*2 (Kleywegt *et al.*, 2001*a* ▶,*b* ▶) and the consistency of NCS pairs. Protein quaternary-structure analysis used the EBI *PISA* server (Krissinel & Henrick, 2007 ▶). Fig. 1 ▶(*b*) was adapted from an analysis using *PDBsum* (Laskowski *et al.*, 2005 ▶). Figs. 1 ▶(*a*) and 2 ▶(*a*) were prepared with *PyMOL* (DeLano Scientific) and Fig. 1 ▶(*c*) was prepared with *ESPript* (Gouet *et al.*, 2003 ▶). Atomic coordinates and experimental structure factors for *Xc*TcmJ from *X. campestris* pv. *campestris* strain ATCC 33913 at 1.6 Å resolution have been deposited in the PDB under accession code 3h50.

## 3. Results and discussion

### 3.1. Overall structure

The crystal structure of *Xc*TcmJ (Fig. 1 ▶ *a*) was determined to 1.6 Å resolution using the MAD method. Data collection, model and refinement statistics are summarized in Table 1 ▶. The final model is comprised of a protein monomer (residues 1–113 and Gly0 from the TEV protease cleavage site), four zinc ions, two acetate molecules and 151 water molecules in the asymmetric unit. As the electron density was not well resolved for some of the side-chain atoms of Lys13, Gln20 and Arg42, these atoms were not included in the structural model. The Matthews coefficient (*V*_M_; Matthews, 1968 ▶) for *Xc*TcmJ is 2.19 Å^3^ Da^−1^ and the estimated solvent content is 43.8%. The Ramachandran plot produced by *MolProbity* shows that 98.2% and 100% of the residues are in favored and allowed regions, respectively.

*Xc*TcmJ is composed of ten β-strands (β1–β10) and one 3_10_-helix (Fig. 1 ▶ *a*). The total β-sheet content is 45.1% and the 3_10_-helical content is 2.6%. The *Xc*TcmJ monomer (Fig. 1 ▶ *a*) contains two antiparallel β-sheets that form a jelly-roll β-sandwich and a topology characteristic of the RmlC-like cupin superfamily (SCOP sunid 51182; Andreeva *et al.*, 2004 ▶). The larger β-sheet has a six-stranded 23(10)581′ topology, while the smaller β-sheet has a four-stranded 4967 topology (Fig. 1 ▶ *a*).

The crystallographic packing of *Xc*TcmJ indicates that a homodimer is the biologically relevant form. This finding is consistent with previous reports (Chin *et al.*, 2006 ▶; Chu *et al.*, 2005 ▶) and with the results of our analytical size-exclusion chromatography coupled with static light scattering. In the homodimer, the N-terminal β-strand β1 of each monomer interacts with β8 in the twofold symmetry-related monomer in a domain-swapped arrangement. The dimer resembles two juxtaposed barrels. One end of each barrel, proximal to the C-­terminus and zinc-binding site, is solvent accessible and forms a putative active site. The two symmetry-related, six-stranded β-sheets in the dimer show a pronounced twist, which enhances structural complementarity to the tightly packed, monomer–monomer interface.

A *DALI* search (Holm *et al.*, 2008 ▶) for entries in the PDB similar to *Xc*TcmJ revealed a multitude of related cupin structures. Alignment of the amino-acid sequence of *Xc*TcmJ with those of related structures is shown in Fig. 1 ▶(*c*). In general, *Xc*TcmJ shares only 10–20% sequence identity with related cupins, even though the structures are quite similar. Many structural homologues contain a bound metal ion, but others do not. An example from the multitude of similar structures is the metal-deficient BH2720 from *Bacillus halodurans* (gi:10175341; PDB code 2oa2), which was reported by the JCSG and which has 28% sequence identity and an r.m.s.d. of 1.46 Å with *Xc*TcmJ for 99 superimposed C^α^ atoms.

### 3.2. The zinc acetate-binding site

**Figure 2:**
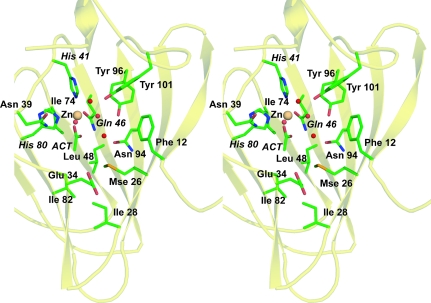
Stereo diagram of residues in the putative active site of *Xc*TcmJ from *X. campestris* pv. *campestris*. The C, O, N and Se (from selenomethionine) atoms of the side chains of *Xc*TcmJ and the bound acetate ligand (labeled ACT) are shown in green, red, blue and orange, respectively. The bound Zn atom and water molecules are shown as orange and red spheres, respectively. His41, Gln46 and His80, which are within coordination distance of the bound zinc, are labeled in italics.

X-ray fluorescence emission spectroscopy on the same *Xc*TcmJ crystal as used for structure determination indicated the presence of selenium and zinc and the absence of other transition metals. To corroborate that zinc is bound at specific sites in the structure and is not just present in the bulk solvent, an anomalous difference map was calculated using the Se remote data (λ_1_ MADSe in Table 1 ▶). The map revealed that, in addition to two electron-dense peaks ∼16σ above the r.m.s. background level at positions corresponding to the Se atoms, other peaks at ∼13σ were found that were not associated with the selenium sites. These peak-height ratios were consistent with the theoretical ratio of Δ*f*′′ for selenium *versus* zinc at λ_1_. One of the bound zinc ions is specifically coordinated to the metal-binding site (Figs. 1 ▶ *a* and 2 ▶), while three other zinc ions are found in crystal-packing interfaces (not shown). The active-site zinc is within co­ordination distance of the side-chain NE2 atoms of two histidines (His41 and His80) and the carboxamide O atom of Gln46. A bound acetate was also identified in an ∼5 Å deep hydrophobic pocket and was most likely acquired from the crystallization solution. The positions of the acetate carboxylate emulates coordination to the zinc by Glu or Asp side chains in other structures. Here, the Glu34 carboxyl is 5 Å from the bound acetate and is too distant for zinc coordination even if it adopted another rotamer (Fig. 2 ▶ *b*).

A bound water molecule, acetate and three side-chain ligands indicate octahedral coordination to the bound zinc. In zinc metalloproteins the metal ion is thought to play either a structural or a catalytic role, with tetrahedral coordination being the most prevalent (McCall *et al.*, 2000 ▶). Structural zinc sites usually contain four protein side-chain ligands (Cys, Glu or Asp) in a tetrahedral coordination, whereas catalytic zinc sites tend to utilize more than four ligands, with one being a water (Auld, 2009 ▶). Thus, the octahedral coordination geometry and coordinating water molecule both suggest a likely functional role for the bound zinc. A list of interactions surrounding the zinc at the putative active site is shown in Table 2 ▶.

Most members of the cupin superfamily typically share two conserved sequence motifs (Dunwell *et al.*, 2001 ▶, 2004 ▶; see Fig. 1 ▶ *c*) that contribute His, Glu and Asp side chains to a conserved metal ion-binding site. An alignment between *Xc*TcmJ and related sequences is shown in Fig. 1 ▶(*c*), with the conserved N-terminal sequence motif highlighted in green and the C-terminal sequence motif in blue. This alignment indicates that, in *Xc*TcmJ, either Asp38 or Asp45 is likely to be a ligand for a bound metal ion. However, the crystal structure shows that the ligand of the metal ion is the carboxamide side chain of Gln46, even though Glu and Asp side chains are more prevalent ligands than the carboxamide side chains of Gln and Asn residues (Hsin *et al.*, 2008 ▶). This type of coordination is also observed in the crystal structure of a related cupin, phosphomannose isomerase from *Candida albicans* (Cleasby *et al.*, 1996 ▶), where an amide side chain is also within coordination distance of the bound metal.

### 3.3. Comparison with apo XC5357

**Figure 3:**
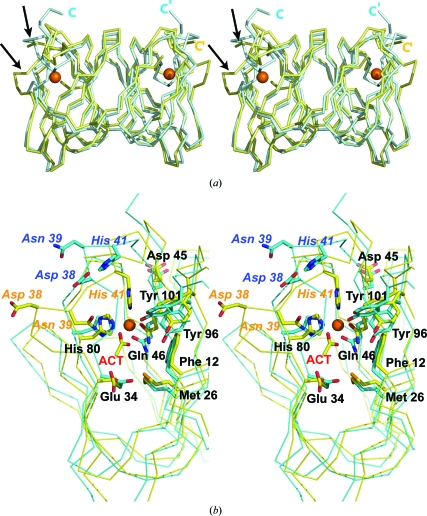
Comparison of the zinc acetate-bound and metal-free (PDB code 2gu9) structures of *Xc*TcmJ from *X. campestris* pv. *campestris*. (*a*). Stereo diagram showing a superposition of the C^α^ traces of the zinc-bound (yellow) and the apo (cyan) forms. The two regions that show significant conformational differences are indicated by arrows. The Zn atom is shown as an orange sphere. (*b*). Stereo diagram of the residues in the putative active site of the zinc acetate-bound structure superimposed on the apo structure (PDB code 2gu9). The C atoms from the side chains and acetate ligand (ACT) of the zinc acetate-bound structure are shown in yellow, while the C atoms of the apo structure are shown in cyan. O, N and Se (SeMet) atoms  in both structures are shown in red, blue and orange, respectively. The Zn atom in the metal ion-bound form is shown as an orange sphere. Those side chains that show significant structural differences in the ligand-bound and the apo states are labeled in italics.

Apo *Xc*TcmJ has previously been reported at 1.4 Å resolution for XC5357 from a different strain of *X. campestris* (PDB code 2gu9; Chin *et al.*, 2006 ▶). Although XC5357 and *Xc*TcmJ share identical amino-acid sequences, their structures show significant differences. XC5357 was crystallized in a triclinic form without zinc, whereas *Xc*TcmJ crystallized in a monoclinic space group in the presence of zinc acetate. Examination of the structures with and without zinc reveals significant rearrangements around the metal-binding site (Figs. 3 ▶ *a* and 3 ▶ *b*). Strand β4 (Gly35–Asp38) in the apo structure is repositioned as a surface loop in the *Xc*TcmJ structure. Additional rearrangements (Asn39–Gly43) in *Xc*TcmJ contribute to the formation of a longer loop segment connecting β4 (Asp32–Gly36) and β5 (Asp45–Asp52) at the open end of the cupin barrel. The conserved His41 is located in one of the loops and the backbone rearrangements in *Xc*TcmJ result in His41 relocating by ∼8 Å to coordinate with the Zn atom near the center of the cupin barrel. In contrast, the other zinc ligands, Gln46 and His80, do not move significantly.

Comparison of the solvent-accessible surfaces (see supplementary material^1^) indicates that a deep surface cavity is eliminated upon the conformational rearrangements arising from the binding of zinc acetate. In the apo structure, this cavity is located between antiparallel β-strands at the C-terminus of each monomer and a loop in the vicinity of His41. In *Xc*TcmJ, this histidine and loop are repositioned upon metal ligation, so that the interactions with the C-­terminal region are lost. One possible explanation for the loss of these interactions is that the crystallized XC5357 contained an eight-residue sequence tag at the C-terminus (Chu *et al.*, 2005 ▶) that was absent from the crystallized *Xc*TcmJ. Consequently, this cavity is replaced by a shallow depression in *Xc*TcmJ and the two-stranded β-­sheet becomes a loop and a small 3_10_ helix. Notwithstanding, both the *Xc*TcmJ and apo structures retain the cavity corresponding to the zinc acetate-binding site. Furthermore, a new channel is formed on the surface of *Xc*TcmJ that is surrounded by nonpolar residues including Ala11, Phe12, Val27 and Ile28 that may reflect the natural substrate-binding site.

### 3.4. Functional implications and conclusions

Extensive investigations have demonstrated the importance of metal ions in the structure and function of diverse cupins (Dunwell *et al.*, 2001 ▶, 2004 ▶). However, it is not understood why some cupins require metal ions for biological activity whereas others do not. Conformational changes induced by metal binding could exert control of reaction specificity or even inhibition of certain biological processes (Babor *et al.*, 2005 ▶; Barondeau & Getzoff, 2004 ▶). From the structural studies alone, it is difficult to define the exact functional implications of metal ion binding to *Xc*TcmJ. However, metal ion binding can have a profound impact on the structure, as illustrated here. Zinc is specifically bound and reorganizes the structure and acetate (likely from the crystallization solution) then serves as a counter ion in the formation of the zinc–protein complex.

Additional information about the protein described in this study is available from TOPSAN (Krishna *et al.*, 2010 ▶)  http://www.topsan.org/explore?PDBid=3h50.

## Supplementary Material

PDB reference: tetracenomycin polyketide synthesis protein (NP_636471.1), 3h50, r3h50sf

Supplementary material file. DOI: 10.1107/S1744309109021988/wd5112sup1.pdf

## Figures and Tables

**Table 1:** Summary of crystal parameters, data collection and refinement statistics for *Xc*TcmJ (PDB code 3h50) Values in parentheses are for the highest resolution shell.

	λ_1_ MADSe	λ_2_ MADSe	λ_3_ MADSe
Space group	*C*2		
Unit-cell parameters (Å, °)	*a* = 48.92, *b* = 72.03, *c* = 32.75, β = 110.76		
Data collection			
Wavelength (Å)	0.9184	0.9796	0.9793
Resolution range (Å)	28.5–1.60 (1.64–1.60)	28.5–1.60 (1.64–1.60)	28.5–1.60 (1.64–1.60)
No. of observations	100153	100180	66773
No. of unique reflections	13913	13898	13763
Completeness (%)	98.6 (91.4)	98.5 (90.3)	98.0 (95.2)
Mean *I*/σ(*I*)	14.0 (2.6)	12.9 (2.4)	10.5 (1.9)
*R*_merge_ on *I*† (%)	11.9 (67.9)	13.1 (73.6)	10.6 (58.8)
Model and refinement statistics			
Resolution range (Å)	28.5–1.60		
No. of reflections (total)	13912		
No. of reflections (test)	696		
Completeness (%)	98.6		
Data set used in refinement	λ_1_		
Cutoff criterion	|*F*| > 0		
*R*_cryst_‡	0.182		
*R*_free_§	0.220		
Stereochemical parameters			
Restraints (r.m.s.d. observed)			
Bond angles (°)	1.42		
Bond lengths (Å)	0.014		
Average isotropic *B* value (Å^2^)	13.1		
ESU¶ based on *R*_free_ (Å)	0.10		
Protein residues/atoms	114/865		
Waters/ions	154/6		

† *R*_merge_ = 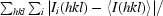 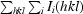.

‡ *R*_cryst_ = 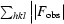 − 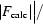 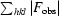, where *F*_calc_ and *F*_obs_ are the calculated and observed structure-factor amplitudes, respectively.

§ *R*_free_ is the same as *R*_cryst_ but for 5.0% of the total reflections chosen at random and omitted from refinement.

¶ Estimated overall coordinate error (Collaborative Computational Project, Number 4, 1994 ▶; Cruickshank, 1999 ▶).

**Table 2:** Short-range contacts to the Zn atom in the putative active site of *Xc*TcmJ

Atom	Interatomic distance (Å)
His41 NE2	2.0
Gln46 OE1	2.1
His80 NE2	2.2
Acetate (ACT) 118 O	2.2
Acetate (ACT) 118 OXT	2.2
Wat181 O	2.3

## Footnotes

1 Supplementary material has been deposited in the IUCr electronic archive (Reference: WD5112).
