# Supplementary material for: Conformational changes associated with the binding of zinc acetate at the putative active site of XcTcmJ, a cupin from Xanthomonas campestris pv. campestris
Source: Acta Crystallogr Sect F Struct Biol Cryst Commun. 2009 Oct 27;66(Pt 10):1347–53. doi: 10.1107/S1744309109021988 (PMC2954225; doi:10.1107/S1744309109021988)
Supplement: Supplementary file 1 [file f-66-01347-sup1.pdf]

SUPPLEMENTARY MATERIAL

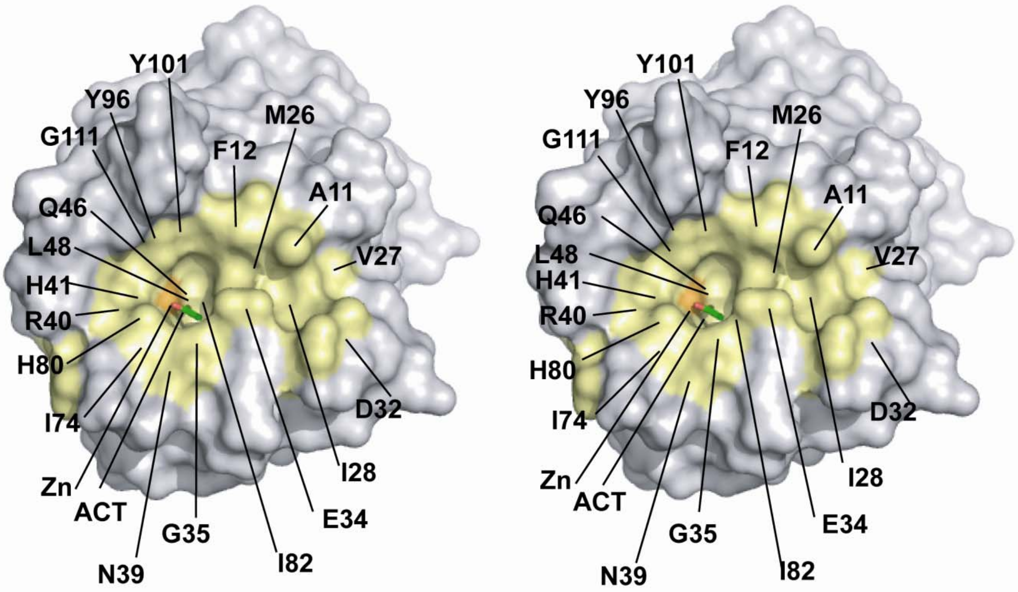

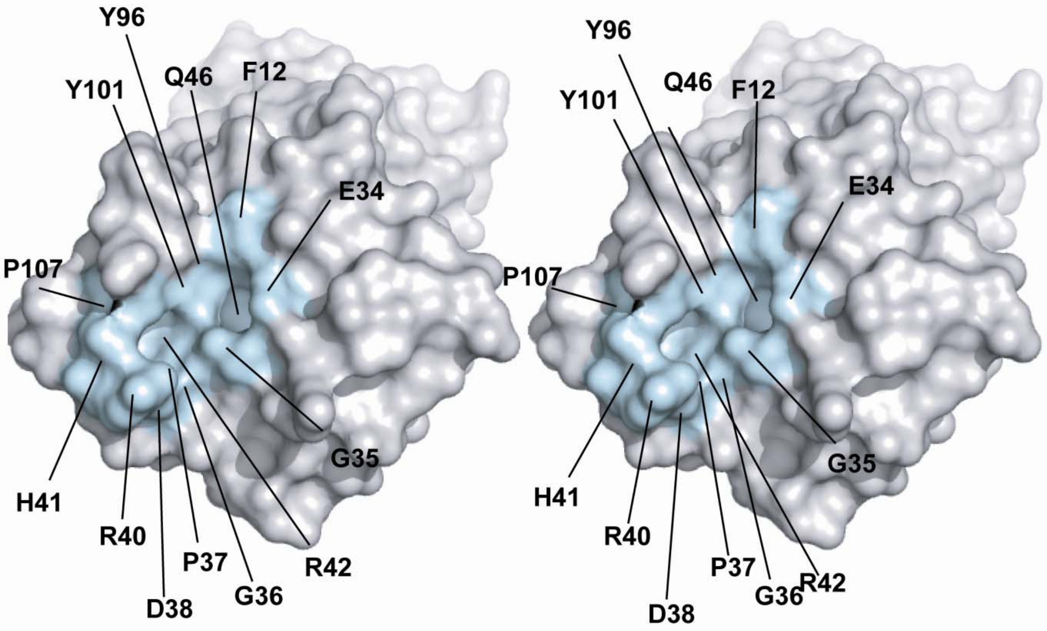

Stereoviews of the active-site cavities on the zinc acetate-bound (top) and apo structure (bottom) of *Xc*TcmJ from *Xanthomonas campestris*. Solvent-accessible surface is shown and was rendered using *PyMOL* (DeLano Scientific). Residues surrounding the cavities were determined with *CASTp* and rendered in yellow for the zinc acetate-bound (top) and cyan for the apo structures with these residues labeled. The surface of the bound zinc (orange) interacting with the acetate (ACT) ligand is shown on the upper surface.
